# Racial disparities in presenting stage and surgical management among octogenarians with breast cancer: a national cancer database analysis

**DOI:** 10.1007/s10549-024-07531-3

**Published:** 2024-11-04

**Authors:** Amulya Vadlakonda, Nikhil L. Chervu, Giselle Porter, Sara Sakowitz, Hanjoo Lee, Peyman Benharash, Nimmi S. Kapoor

**Affiliations:** 1 Department of Surgery, University of California, 15503 Ventura Blvd, Ste. 150, Encino, Los Angeles, CA 91436 USA; 2 Department of Surgery, Harbor-UCLA Medical Center, Torrance, CA USA

**Keywords:** Racial disparities, Breast cancer, Elderly, Screening, Breast cancer treatment, Social determinants of health

## Abstract

**Background:**

As the US faces a diverse aging population, racial disparities in breast cancer outcomes among elderly patients remain poorly understood. We evaluate the association of race with presenting stage, treatment, and survival of invasive breast cancer among octogenarians.

**Methods:**

Women (≥ 80 years) with invasive breast cancer were identified in 2004–2020 NCDB. To facilitate comparison, only non-Hispanic Black and non-Hispanic White patients were included; patients of Hispanic ethnicity were excluded. Demographics, tumor characteristics, and treatments were assessed by race. Overall survival was compared using the logrank test. Multivariable logistic and Cox proportional hazard regression models were developed to evaluate the independent association of race with outcomes of interest.

**Results:**

Of 222,897 patients, 19,059 (8.6%) were Black. Most patients had stage I ER + HER2- invasive ductal carcinoma. Black patients more frequently had greater comorbidities, low income and education, and advanced stage (*p* < 0.001 each; ref: White). Following adjustment, Black women had increased likelihood of Stage III/IV over time, as well as increased odds of chemotherapy (AOR 1.22, 95% CI 1.15 – 1.29) and non-operative management (AOR 1.82, 95% CI 1.72 – 1.92; ref: White). Although Black patients had lower survival rates compared to White, race was not associated with 5-year mortality following adjustment for stage, receipt of surgery, and adjuvant treatments (*p* = 0.34).

**Conclusions:**

Inferior survival among elderly Black patients appears be driven by advanced stage at presentation. While such disparities are narrowing in the present era, future work must consider upstream interventions to ensure equitable outcomes for all races.

## Introduction

Breast cancer remains the leading cause of cancer-related death among women in the United States, with Black patients experiencing nearly 50% greater mortality compared to White counterparts [1]. As the medical and surgical treatments for breast cancer have evolved over the last two decades, survival among White women has increased significantly; however, such improvements have not been achieved for Black women [2]. Prior literature has hypothesized this disparity to be, in part, attributable to delays in diagnosis caused by reduced access to screening services among Black patients [3–5].

At the same time, the risk of developing breast cancer increases with advancing age; however, there is controversy regarding the role of screening mammography in older individuals [6]. Recent guidelines from the United States Preventive Services Task Force reflect a lack of high quality evidence to support such screening in patients older than 75 years of age due to the risk of overtreatment [7]. Furthermore, both the National Comprehensive Cancer Network (NCCN) and Society of Surgical Oncology via its ‘Choosing Wisely Campaign’ have recommended for de-escalation of screening and treatment in older patients [8, 9]. Specifically, the NCCN recommends screening mammography up to the age of 74, and the Choosing Wisely Campaign advises against routine sentinel lymph node biopsy. Regardless of these changes in guidelines, racial differences in screening, diagnosis, and treatment of breast cancer are increasingly evident among older patients [10, 11]. The impact of such racial differences on treatment and survival of invasive breast cancer among elderly patients remains understudied.

Thus, the present study utilized the National Cancer Database (NCDB) to investigate racial disparities in the presentation, treatment, and survival of invasive breast cancer among octogenarians. While racial disparities in breast cancer care, access, and outcomes are well-documented, we believe that the negative synergistic effect of increased age may magnify these differences. We therefore hypothesized that, among octogenarians, that Black race would be associated with advanced clinical stage of breast cancer at diagnosis and increased hazard of mortality.”

## Methods

This was a retrospective cohort study of the 2004–2020 National Cancer Database (NCDB). This database is jointly overseen by the American College of Surgeons Commission on Cancer (CoC) and the American Cancer Society. With records from over 1,500 accredited centers, the NCDB comprises approximately 70% of US cancer patients [12].

**Fig. 1:**
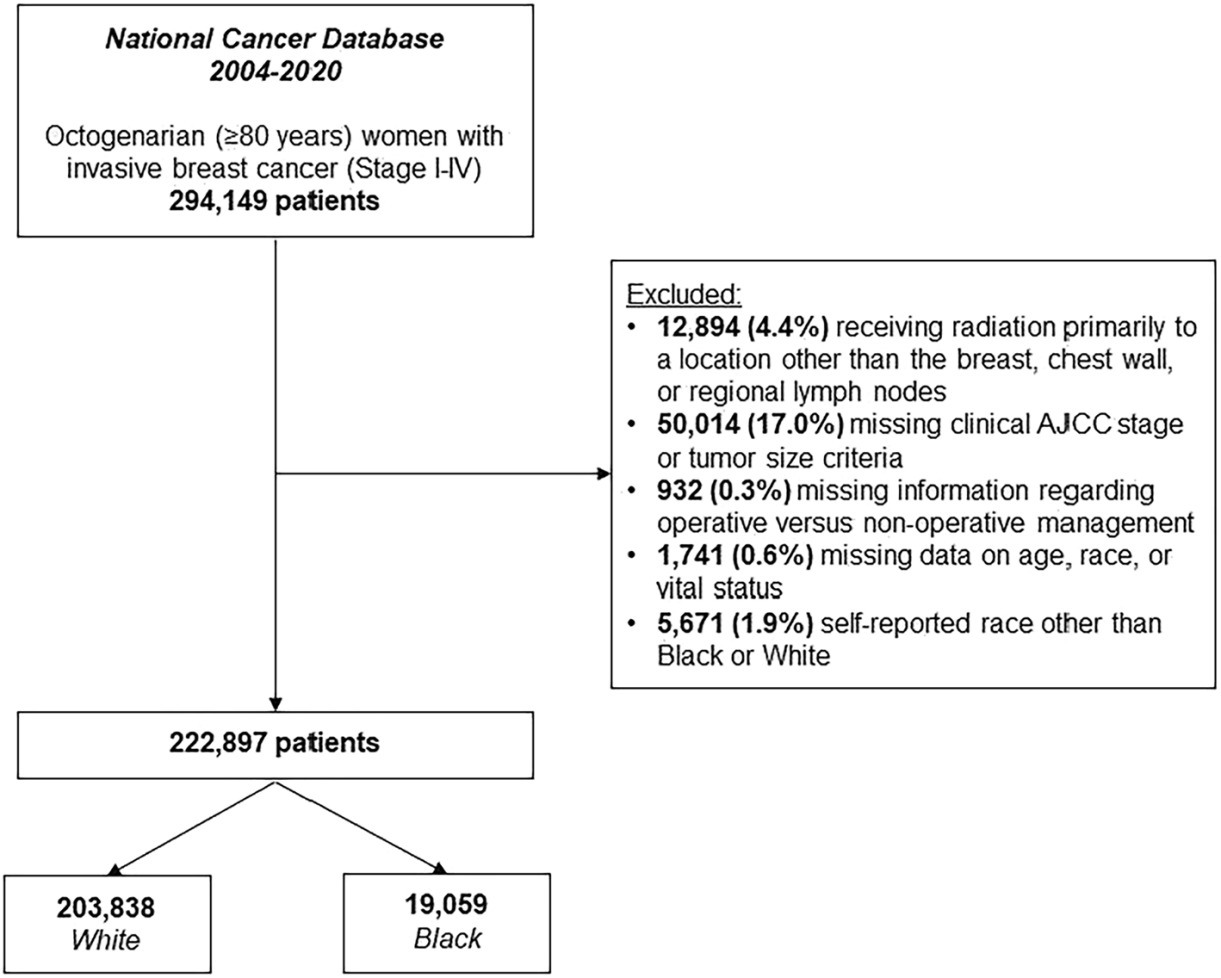
Exclusion criteria

Octogenarian (≥ 80 years) women with invasive breast cancer were identified in the 2004–2020 NCDB. Only ductal, lobular, or mixed histological subtypes were included for analysis, as denoted by relevant codes from the *International Classification of Disease-Oncology, Third Revision* (ICD-O-3). Records missing data regarding operative management, histology, overall clinical stage, demographic data, or vital status were excluded from analysis (Fig. 1). Due to the relatively small sample size of patients from other races, only those with a self-reported race of White or Black were included. The NCDB does not report Hispanic ethnicity from race; therefore, White and Black race were exclusive of Hispanic ethnicity.

All patients and hospital factors were defined in accordance with the NCDB data dictionary. Notably, the burden of comorbidities in the cohort was quantified by the Charlson-Deyo Index (CDI), as given by the NCDB. Clinical and pathological stages were assigned using the 8th edition of the American Joint Committee on Cancer (AJCC) Staging System. To ensure equivalent comparisons by stage, patients diagnosed prior to 2018 were re-classified by the tumor size criteria of the 8th edition. AJCC clinical stages III and IV were considered late-stage.

The primary endpoints of the study were stage at diagnosis, operative management, sentinel lymph node biopsy (SLNB), axillary lymph node dissection (ALND), and receipt of systemic therapies. Additionally, overall survival was assessed at 1, 3, and 5 years among patients with non-metastatic disease only.

Temporal trends were assessed using a non-parametric test across ordered groups (nptrend) [13]. The significance of intergroup differences of demographic, clinical, and hospital factors, were evaluated using Pearson’s χ^2^ and adjusted Wald tests, as appropriate. The log-rank test was utilized to assess the difference in unadjusted survival rates by race, which were graphically depicted using the Kaplan–Meier method. Multivariable logistic regression and Cox proportional hazard models were constructed to evaluate the independent association of patient race with outcomes of interest. Covariates were selected by clinical relevance and with guidance by the Least Absolute Shrinkage and Selection Operator (LASSO). Briefly, LASSO is an automated regularization algorithm that minimizes collinearity and overfitting to enhance the out-of-sample reliability of the model [14]. Models were evaluated and optimized using Bayesian information criteria and receiver operating characteristics.

Categorical variables are shown as proportions (%), while continuous variables are reported as means with standard deviation or medians with interquartile range, as appropriate. Outputs of logistic regression models are reported as adjusted odds ratios (AOR), while the Cox proportional hazard model outputs are shown as hazard ratios (HR), both with 95% confidence intervals (CI). Statistical significance for all analyses was considered at *α* < 0.05. Stata 18.0 software (StataCorp LP, College Station, TX) was used for all statistical analysis. This study was deemed exempt from full review by the Institutional Review Board at the University of California, Los Angeles, due to the de-identified nature of the data.

## Results

### Trends and characteristics of elderly black and white patients

**Fig. 2:**
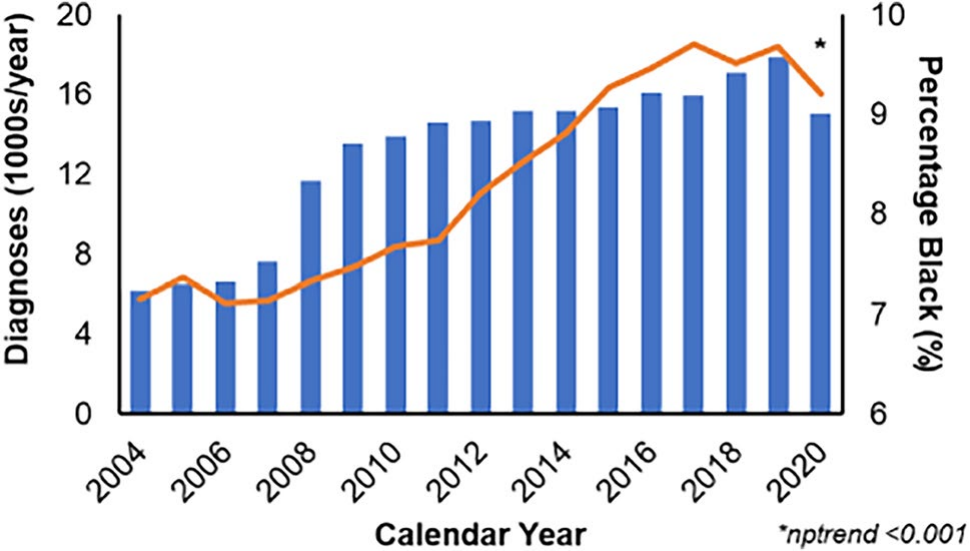
National trends in the diagnosis of invasive breast cancer among octogenarians

Of 222,897 octogenarian patients, 19,059 (8.6%) were Black. Most patients had stage I ER + HER2- invasive ductal carcinoma. The proportion of these patients who self-identified as Black increased significantly from 7.1% in 2004 to 9.2% in 2020 (nptrend < 0.001; Fig. 2). Compared to White patients, Black patients were more likely to have a higher Charles-Deyo Comorbidity Index (p < 0.001).

### Baseline characteristics and unadjusted outcomes

Black patients more commonly were in the lowest quartile of household income (52.8 vs 17.2%, *p* < 0.001) and education (49.7 vs 18.0%, *p* < 0.001). Black patients were more frequently from metropolitan areas (90.9 vs 85.3, *p* < 0.001), and treated at academic hospitals (37.0 vs 21.6%, *p* < 0.001). Compared to White patients, Black women were less likely to have lobular cancer (10.1 vs 12.4%, *p* < 0.001), and more likely to be diagnosed with triple negative breast cancer (TNBC; 15.5 vs 8.8%, *p* < 0.001; Table 1). Black patients were more frequently diagnosed with Stage III (11.1 vs. 7.2%) and Stage IV disease (9.5 vs 6.4%, both p < 0.001).

**Table 1:** Demographic, clinical, and hospital characteristics of octogenarian patients with breast cancer. *IQR, interquartile range; *AJCC*; American Joint Commission on Cancer

	White (*n* = 203,838)	Black (*n* = 19,059)	p
Age, years (median, IQR)	84 [81–87]	84 [81–87]	< 0.001
Charlson-deyo comorbidity Index, %			< 0.001
0	74.6	66.5	
1	16.9	20.7	
2	5.5	7.0	
3	3.0	5.7	
Income quartile, %			< 0.001
76-100th	30.3	11.9	
51-75th	22.6	12.4	
26-50th	20.7	17.5	
0-25th	15.8	46.7	
Unknown	10.6	11.5	
Payer, %			< 0.001
Private	7.4	8.9	
Medicare	90.1	84.3	
Medicaid	0.9	2.6	
Not Insured	0.3	0.6	
Other^a^	0.4	0.3	
Unknown	1.1	1.3	
Education quartile, %			< 0.001
76-100th	19.1	4.7	
51-75th	29.2	13.8	
26-50th	24.8	26.1	
0-25th	16.4	44.0	
Unknown	10.6	11.5	
Rural status, %			< 0.001
Metropolitan	83.5	89.7	
Urban	12.9	7.8	
Rural	1.8	1.2	
Unknown	1.8	1.3	
AJCC clinical stage, %			< 0.001
Stage I	56.4	44.2	
Stage II	30.1	35.3	
Stage III	7.2	11.1	
Stage IV	6.4	9.5	
Histology, %			< 0.001
Ductal	68.2	68.8	
Lobular	12.4	10.1	
Mixed	4.9	3.5	
Other	14.4	17.6	
Receptor status, %^b^			< 0.001
ER + or PR + / HER2-	75.1	66.9	
Triple Negative	8.8	15.6	
HER2 +	8.7	9.2	
Unknown	7.4	8.3	
Hospital region, %			< 0.001
Northeast	24.0	20.3	
Midwest	27.9	22.5	
South	31.4	51.2	
West	16.6	6.0	
Hospital type, %			< 0.001
Academic	22.1	37.5	
Community	55.8	40.7	
Integrated network	22.2	21.8	

^a^As defined by the National Cancer Database data dictionary

^b^Of all patients from 2010–2020 (White: *n* = 142,732, Black: *n* = 14,194), as HER2 status was recorded in the NCDB first in 2010

Black women less frequently received mastectomy (16.5 vs 18.3%) or breast-conserving surgery (41.3 vs 52.5%, both p < 0.001). Among those receiving mastectomy, however, Black patients more commonly underwent modified radical mastectomy (MRM) (38.7 vs 32.6%, *p* < 0.001), relative to White women. A negligible proportion of the entire population had radical mastectomy (0.3%). Even among only Stage I-II patients, Black patients remained more likely to receive non-operative management (21.8 vs 13.3%) and less likely to receive breast-conserving surgery (50.2 vs 59.3%, both *p* < 0.001; Table 2). Furthermore, Black patients with Stage I-III breast cancer were less likely to undergo lymph node intervention and radiation, compared to stage-matched White counterparts. Nonetheless, among both metastatic and non-metastatic patients, Black women remained more likely to undergo chemotherapy (Table 2).

**Table 2:** Unadjusted comparison of receipt of operation, therapies, node intervention between White and Black octogenarian women, stratified by AJCC clinical stage. *AJCC, American Joint Commission on Cancer; SLNB, sentinel lymph node biopsy; ALND, axillary lymph node dissection

		Stage I			Stage II			Stage III			Stage IV	
	White (114,870)	Black (8,421)	p	White (61,309)	Black (6,721)	p	White (14,681)	Black (2,112)	p	White (12,978)	Black (1,805)	p
Axillary Surgery, %^a^			< 0.001			0.001			0.001			0.19
No	38.6	42.0		37.5	40.7		47.6	51.4		82.9	84.2	
Yes	61.4	58.0		62.5	59.3		52.4	48.6		17.1	15.8	
Among 2012–2020, %												
SLNB	47.4	44.0	< 0.001	34.8	30.0	< 0.001	14.3	14.5	0.87	10.9	11.3	0.64
ALND	5.7	6.0	0.39	15.4	18.4	0.87	28.7	27.7	0.47	5.5	5.3	0.76
Operation, %			< 0.001			< 0.001			< 0.001			0.003
Non-operative	9.9	16.1		19.8	28.9		41.7	50.8		85.1	88.2	
Breast conserving surgery	69.6	62.2		40.1	35.1		12.8	9.7		5.4	3.9	
Simple mastectomy	16.0	16.5		25.6	20.9		17.8	13.8		4.5	3.1	
Modified radical mastectomy	4.4	5.0		14.1	14.8		26.8	24.7		4.8	4.6	
Radical mastectomy	0.1	0.2		0.4	0.4		1.0	1.0		0.3	0.2	
Chemotherapy, %	3.1	4.7	< 0.001	9.2	11.3	< 0.001	20.7	23.4	0.004	20.1	22.4	0.02
Timing, %^b^			0.71			< 0.001			0.26			0.22
Neoadjuvant	18.3	19.0		40.9	48.4		75.2	77.5		88.3	90.4	
Adjuvant	81.7	81.0		59.1	51.6		24.8	22.5		11.7	9.6	
Hormone Therapy, %	56.2	56.8	0.60	58.9	55.9	< 0.001	56.8	52.5	< 0.001	51.0	44.8	< 0.001
Timing, %^c^			< 0.001			< 0.001			0.001			0.005
Neoadjuvant	16.9	20.9		33.8	41.8		61.9	67.1		88.3	91.6	
Adjuvant	83.1	79.1		66.2	58.2		38.1	32.9		11.7	8.4	
Immunotherapy, %	1.3	1.7	0.02	3.3	3.4	0.83	5.4	6.3	0.09	6.8	6.2	0.33
Radiation, %	31.5	29.3	< 0.001	26.6	24.0	< 0.001	23.7	21.3	0.01	2.7	2.3	0.38

^a^Prior to 2012, the NCDB only reported the receipt of either SLNB or ALND. For patients diagnosed after 2012, the proportion of patients receiving each of these therapies are reported individually below

^b^Among patients who received chemotherapy

^c^Among patients who received hormone therapy

**Fig. 3:**
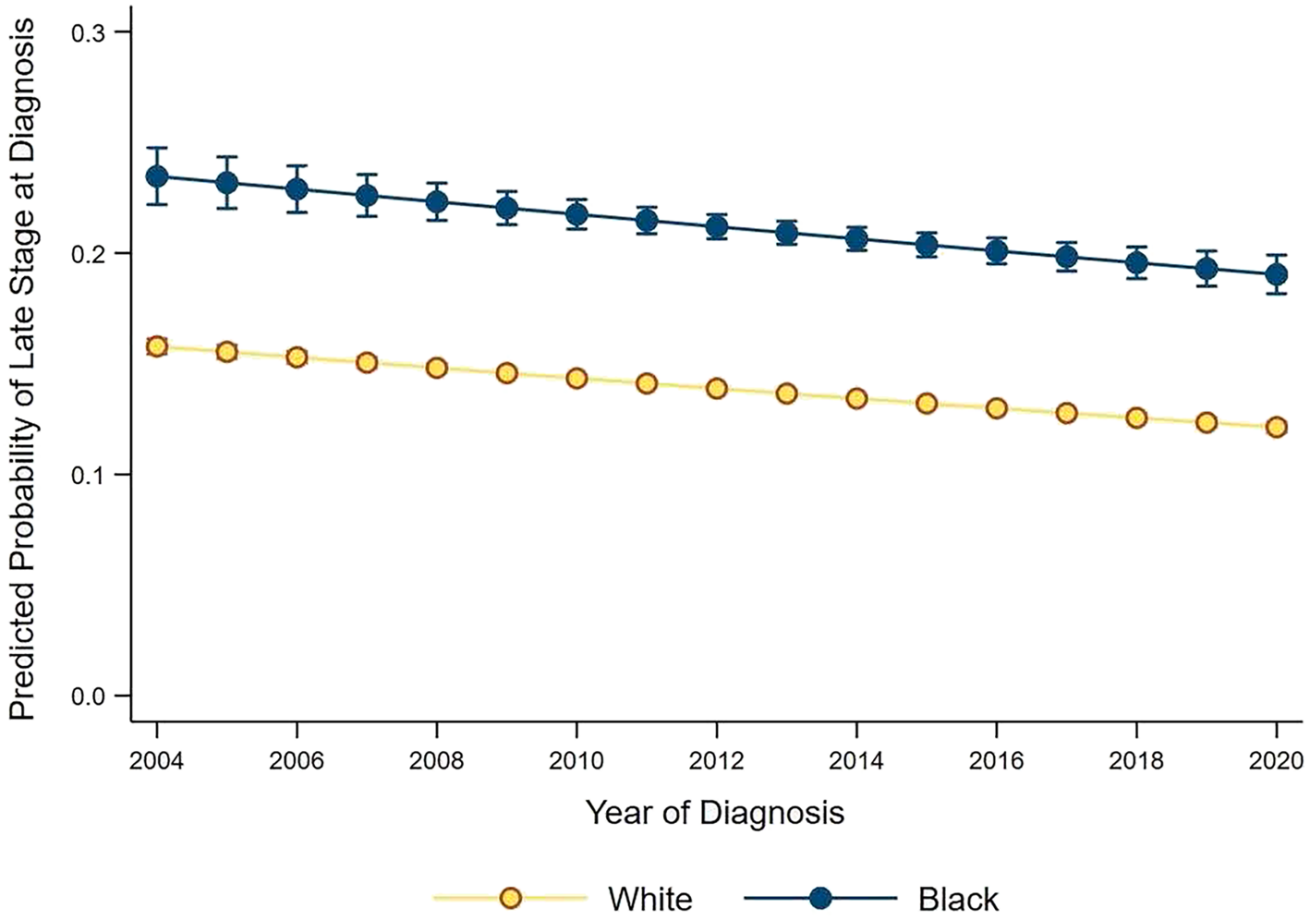
Overall survival stratified by race and stage

All patients had a median follow up time of 43.9 months. Among survivors, Black women had shorter follow up than White women (48.4 vs 52.2 months, *p* < 0.001). Black patients had lower rates of overall survival at 1 (90.0 vs 92.0%, *p* < 0.001), 3 (69.7 vs 74.8%, *p* < 0.001), and 5 years (53.8 vs 58.6%, *p* < 0.001), relative to White patients. However, with further stratification of patients by stage at diagnosis, the differences in survival by race narrowed considerably (Fig. 3).

### Risk-adjusted outcomes

**Fig. 4:**
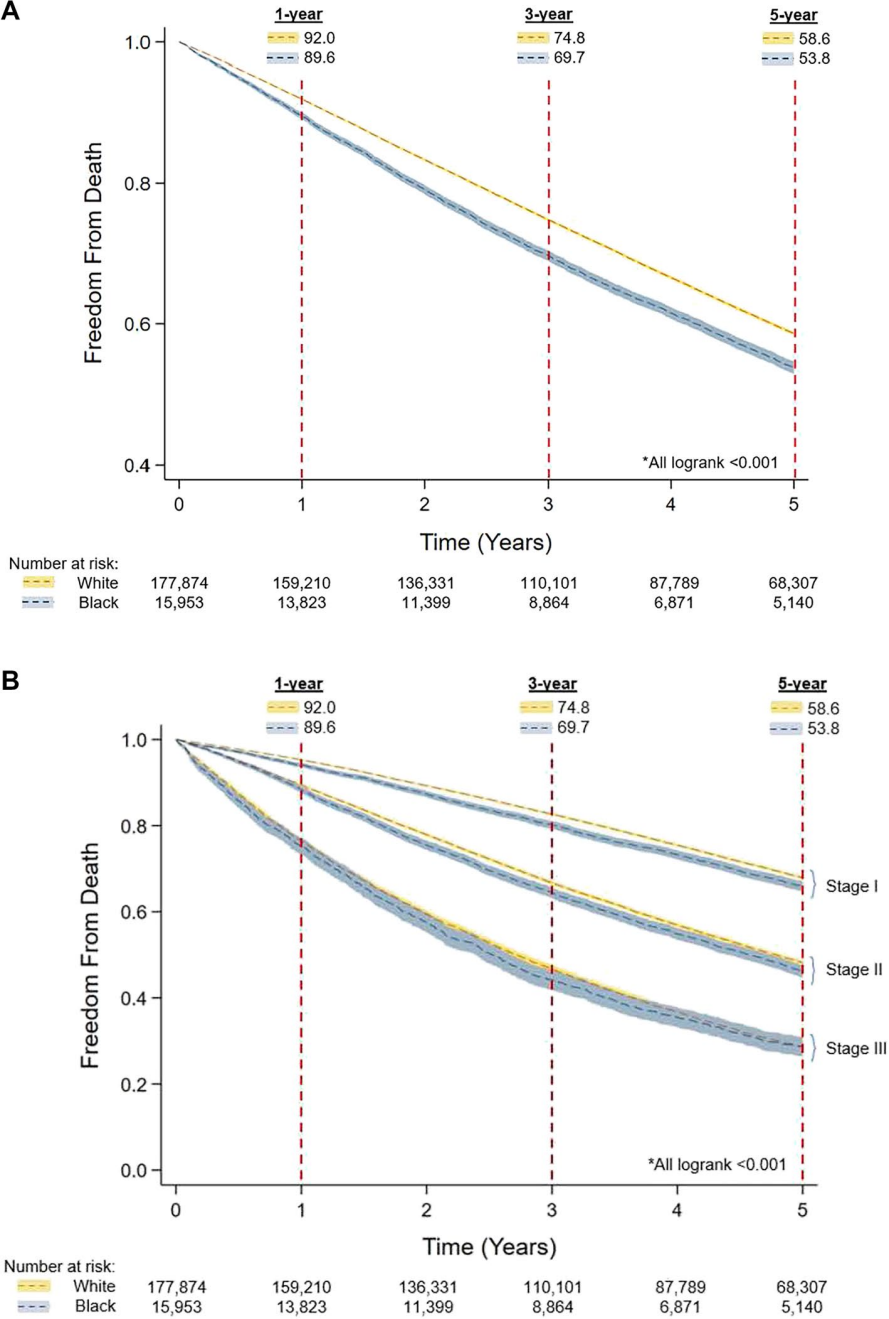
Trends in predicted probability of late-stage diagnosis from 2004 to 2020 by race

The risk-adjusted probability of late-stage diagnosis is decreasing for both Black and White patients from 2004 to 2020 (Fig. 4). However, Black race was independently associated with late-stage at diagnosis (AOR 1.47, 95% CI 1.41 – 1.54). All factors included for multivariable adjustment are shown in the complete model (Table 3). The interaction term between patient race and year of diagnosis was not significant, thereby demonstrating a continued disparity independent of time at diagnosis (*p* = 0.55).

**Table 3:** Multivariable logistic regression model to investigate the factors associated with late-stage at diagnosis, defined as AJCC clinical stage III or IV. The model was constructed with adequate discrimination (c-statistic = 0.70). *AJCC, American Joint Committee on Cancer; AOR, adjusted odds ratio; CI, confidence interval

	AOR	95% CI	P
Black race (ref: White)	1.47	1.41 – 1.54	< 0.001
Age, years (median, IQR)	1.06	1.06 – 1.07	< 0.001
Charlson-deyo comorbidity index, %			
0	1	(Ref)	–
1	1.13	1.09 – 1.17	< 0.001
2	1.33	1.26 – 1.40	< 0.001
3	1.43	1.33 – 1.53	< 0.001
Income quartile, %			
76-100th	1	(Ref)	–
51-75th	0.97	0.93 – 1.01	0.11
26-50th	0.97	0.93 – 1.02	0.23
0-25th	0.98	0.93 – 1.03	0.44
Payer, %			
Private	1	(Ref)	–
Medicare	1.12	1.06 – 1.17	< 0.001
Medicaid	1.29	1.13 – 1.47	< 0.001
Not Insured	2.10	1.71 – 2.57	< 0.001
Other^a^	0.98	0.76 – 1.25	0.86
Education quartile, %			
76-100th	1	(Ref)	–
51-75th	1.10	1.05 – 1.14	< 0.001
26-50th	1.20	1.14 – 1.26	< 0.001
0-25th	1.32	1.25 – 1.40	< 0.001
Rural status, %			
Metropolitan	1	(Ref)	–
Urban	0.96	0.92 – 1.01	0.08
Rural	0.95	0.86 – 1.06	0.36
Histology, %			
Ductal	1	(Ref)	–
Lobular	1.08	1.03 – 1.12	< 0.001
Mixed	0.74	0.69 – 0.80	< 0.001
Other	1.43	1.38 – 1.48	< 0.001
Laterality, %			
Unilateral	1	(Ref)	–
Bilateral	9.67	6.69 – 13.97	< 0.001
Unknown	10.45	8.88 – 12.30	< 0.001
Primary Site, %			
Nipple	1	(Ref)	–
Central	0.85	0.74 – 0.98	0.02
Upper inner quadrant	0.26	0.23 – 0.30	< 0.001
Lower inner quadrant	0.36	0.31 – 0.42	< 0.001
Upper outer quadrant	0.42	0.36 – 0.47	< 0.001
Lower outer quadrant	0.42	0.36 – 0.48	< 0.001
Axillary	1.10	0.90 – 1.35	0.35
Overlapping	0.53	0.46 – 0.61	< 0.001
Not otherwise specified	1.81	1.59 – 2.08	< 0.001
Hospital region, %			
Northeast	1	(Ref)	–
Midwest	0.96	0.82 – 0.99	0.04
South	0.87	0.83 – 0.90	< 0.001
West	0.86	0.82 – 0.90	< 0.001
Hospital region, %			
Academic	1	(Ref)	–
Community	0.97	0.94 – 1.01	0.08
Integrated network	0.97	0.93 – 1.01	0.18
Year of diagnosis (per year)	1.00	0.98 – 1.00	0.90

### Receipt of operation, lymph node intervention, radiation, and systemic therapies

Following multivariable risk adjustment among only non-metastatic patients, Black race was independently associated with non-operative management (AOR 1.82, 95% CI 1.72 – 1.92). This model included age, Charlson-Deyo Comorbidity Index, income quartile, education quartile, payer, rural status, receptor status, tumor histology, laterality, receipt of radiation, chemo, hormone therapy, immunotherapy, and hospital region. Black race was additionally associated with lower odds of lymph node intervention (AOR 0.82, 95% CI 0.79 – 0.85), but greater odds of chemotherapy (AOR 1.22, 95% CI 1.15 – 1.29). Black race did not significantly alter the odds of radiation therapy after risk adjustment (p = 0.56).

### Risk-adjusted survival among non-metastatic patients

After multivariable risk adjustment, Black race was significantly associated with a greater hazard ratio of mortality at 3 (HR 1.05, 95% CI 1.01 – 1.08), and 5 years (HR 1.03, 95% CI 1.01 – 1.05). Upon the additional adjustment of both stage and receipt of surgery in the Cox proportional hazard model, Black race was no longer associated with mortality at 3 (*p* = 0.09) and 5 years (*p* = 0.34). However, late-stage diagnosis was associated with greater hazard ratio of death (3 years: HR 1.97, 95% CI 1.92 – 2.03, 5 years: HR 1.89, 95% CI 1.84 – 1.93), relative to early-stage diagnosis for both Black and White patients. Receipt of surgery was linked to a decreased hazard ratio of mortality at 3 (breast-conserving surgery: HR 0.34, 95% CI 0.33 – 0.35; simple mastectomy: HR 0.42, 95% CI 0.41 – 0.44; MRM: HR 0.60, 95% CI 0.57 – 0.62) and 5 years (breast-conserving surgery: HR 0.39, 95% CI 0.38 – 0.40; simple mastectomy: HR 0.48, 95% CI 0.47 – 0.49; MRM: HR 0.63, 95% CI 0.61 – 0.65).

## Discussion

Using data from the NCDB from 2004 to 2020, we investigated the role of race in the presenting stage, treatment, and survival of breast cancer amongst octogenarians. Notably, we observed the incidence of breast cancer among older individuals to be decreasing across the study period, which may indicate improvements in the screening and detection of breast cancer over time. We further noted Black women to be more likely to have aggressive tumor subtypes and advanced stage at presentation, relative to White women. Although Black patients were found to have inferior overall survival compared to White counterparts, the association between race and mortality was found to be insignificant after the adjustment of stage at presentation, receipt of surgery, and adjuvant therapies. With implications for the evolving guidelines regarding the screening and treatment of invasive breast cancer in octogenarian women, several of these findings warrant further discussion.

Important differences were noted between clinical features of breast cancer presentation between White and Black patients. Previous literature has established Black women to have a greater risk of aggressive TNBC and younger age at presentation [15–17]. Similarly, in the present study, we found Black patients to more frequently present with aggressive tumor subtypes such as TNBC breast cancer. Additionally, Black women faced higher rates of late-stage breast cancer diagnosis that persisted over time. Newman and colleagues have postulated such differences to be a product of both hereditary patterns of susceptibility and complex socioeconomic dynamics, including poverty, access to regular healthcare, and early utilization of diagnostic biopsy [18]. Similarly, we demonstrate Medicaid insurance, uninsured status, increased index of comorbidities, and lowest education quartile to be associated with advanced stage at diagnosis. In a study of over 175,000 patients, Ko et al. found racial disparities in advanced stage at presentation to be mediated by Medicaid insurance and uninsured status, suggesting interplay between socioeconomic status, access to healthcare, and race [19]. Our findings underscore that while inherent differences in tumor biology may contribute to differences by race, it remains crucial to address the social determinants of health which underlie and exacerbate racial disparities in the diagnosis of breast cancer.

Regardless of stage at diagnosis, however, Black patients were more likely than White patients to be managed nonoperatively. When surgery was employed, Black patients were more likely to undergo more extensive surgery, with ALND instead of SLN biopsy, or mastectomy instead of breast conserving surgery. When controlling for multiple variables including clinical stage and Charlson-Deyo Comorbidity Index, among other factors, Black patients remained 82% more likely than White patients to have nonoperative management. Our findings are consistent with a 2007 retrospective analysis by Lund and colleagues, which demonstrated Black patients to be less likely to receive breast-cancer-directed surgery than White counterparts, with lower rates of radiation with breast-conserving surgery among those operatively managed [20]. There are also growing, simultaneous concerns of over- and undertreatment in the elderly which may lead to varying treatment practices in this population [21, 22]. However, a recent NCDB study found that patients older than 80 years are likely to benefit from stage-appropriate surgical intervention [23]. In light of such data, our findings emphasize the nonstandard and potentially low value treatment of elderly Black patients with invasive breast cancer, which has persisted over time.

Considerable literature has demonstrated Black patients to have inferior survival following breast cancer diagnosis, relative to White counterparts [24, 25]. We similarly demonstrate elderly Black patients to have lower survival at 3 and 5 years, relatively to octogenarian White women. However, such disparities appear to be driven by stage at presentation and receipt of surgery. Following adjustment for such factors, the independent association between patient race and mortality was no longer significant, suggesting racial disparities affecting stage at diagnosis and surgical treatment to be most significant in affecting outcomes. Specifically, differences in regular preventative maintenance and transportation may result in significant disadvantage for Black women, compared to their White counterparts [26]. Prior research has demonstrated that Black women have significantly higher rates of disability above the age of 60, compared to White women, mostly due to chronic disease [27]. Black patients, nevertheless, have lower rates of healthcare utilization in terms of regular physician visits, annual vaccination, mammograms, and other scheduled screening [26, 28]. Difficulties in transportation likewise disproportionately affect the Black population. Park et al. found that Black rural patients report significantly higher rates of transportation difficulty [29]. Notably, lower income was the only studied factor associated with higher rates of transportation difficulty for White patients. Increasing age and female sex, on the other hand, were associated with higher rates of transportation difficulty among Black patients [29]. Given these factors, our results may point to the complex multifaceted care required for effective diagnosis and treatment of breast cancer. Future work should examine local and system-wide policies to ensure timely diagnosis as well as case management strategies to ameliorate racial disparities in this area.

This study has several limitations. First, we are unable to conclude any causal relationship between race and outcomes of interest, due to the retrospective and observational nature of the study. Furthermore, the NCDB is a large national database and is thus subject to coding errors and missing values. The database does not collect information about clinical and surgical decision making, imaging studies, breast-cancer specific survival, cause of death, or personal and cultural factors which may affect race-based differences in utilization of treatments. We thus cannot adequately control for selection bias due to surgeon selection bias, low-stage or indolent disease prompting nuanced shared decision-making, or patient comorbid conditions that may affect surgical risk. Moreover, current literature has demonstrated higher breast cancer surgery refusal rates among Black patients that cannot be determined adequately within the NCDB [30]. We were likewise unable to ascertain whether patients underwent screening mammography at the recommended ages. As noted in the Methods section, we only compared non-Hispanic Black patients to non-Hispanic White patients. Although the NCDB has been shown to account for more than 70% of nationwide cancer cases, it noticeably underreports cases for Asian/Pacific Islander, American Indian/Alaska Native, and Hispanic populations [31]. We therefore believe that this choice was necessary to enhance population comparison, but may limit the overall applicability of our findings. Furthermore, we are unable to account for patients receiving treatment at non-CoC-accredited institutions. Given known-disparities in healthcare access, our data may have a relative overrepresentation of non-Hispanic White patients, compared to Black patients. Nonetheless, we use robust statistical methods to investigate the current landscape of racial disparities in diagnosis and treatment of invasive breast cancer in the elderly.

While the incidence of late-stage breast cancer diagnosis is decreasing over time, Black octogenarians continue to present with more aggressive late-stage tumors than White patients, and are less likely to undergo any surgical intervention regardless of stage. To equalize presenting stage of diagnosis and thus improve survival, upstream interventions should be implemented to avoid adverse consequences for Black patients.

## Author contributions

AV wrote the first draft, collected, and analyzed the data. NLC assisted with data analysis and revised the manuscript. SS, GP, HL and PB assisted with data acquisition, figure design, and analysis. NSK designed the study, revised the manuscript, and supervised the project. All authors reviewed the manuscript.

## Data Availability

Data from this study can be found in the National Cancer Database at https://www.facs.org/quality-programs/cancer-programs/national-cancer-database/.
